# Oral health care at major sporting competitions

**DOI:** 10.1038/s41415-025-9004-9

**Published:** 2026-02-27

**Authors:** Wendy Turner, Peter Fine

**Affiliations:** 308962528097595151999 Queens´s University Belfast, Belfast, United Kingdom; 312221935833284298858 UCL Eastman Dental Institute, London, United Kingdom

## Abstract

Medical facilities at major sporting events can have a huge influence on the overall enjoyment and success of the event for both athletes and spectators. The inclusion of dental care facilities within the medical team is desirable following the clear link between oral health and athletic performance, prevention of dental trauma and keeping athletes pain free during competition. Competition medical care organisers should discuss oral healthcare provision at an early stage of development with their oral health lead, having appointed an oral health lead at an early stage. The role of the dentist is twofold: general dentists who volunteer to facilitate the dental suite at the medical centre and undertake routine dental care of athletes, and specialist sports dentists who work within the field of play at vulnerable sports to deal with orofacial and dental trauma. Both groups of dentists show great skill, huge enthusiasm and a large degree of determination to support the athletes. The value of having dentists available at major sporting events is becoming increasing recognised by sports medics who appreciate the specialist knowledge and skills that the dental professionals bring to the team.

## Key points

- Having dentists as members of the medical team at major sporting events has been shown to be supportive, important and educational.
- The role of the sports dentist at major sporting events in dealing with field-of-play orofacial injuries adds to the excellent support structures in place for elite athletes.
- Competent sports dentists can resolve both hard and soft tissue injuries on site, resulting in a speedy return to play for many of the athletes.

## Introduction

The opportunity for elite athletes to travel the world for competitions has become a common occurrence over recent years as major sporting events are now hosted by countries from all continents, including developing countries, where resources may be restricted, allied healthcare professional services may be limited, and transportation and other facilities may be insufficient.^1^ All sporting events have inherent risks of trauma and injury to athletes which may have a devastating impact on their sporting performance.^2^^,^^3^^,^^4^ There is also a significant risk from existing medical conditions, for example cardiac illness, which could be devastating to an elite athlete.^5^ The link between poor oral health and heart disease is well-established.^6^ During the 1996 Olympic Games (Atlanta, USA), an estimated five million people (including visitors and residents), from 197 different countries gathered for the games. This necessitated unparalleled medical planning, which lay the foundations for medical cover at future major sporting events.^7^ While dental treatment at previous Olympic Games had been undertaken and recorded, it was not until the London 2012 Olympic Games that research into the oral health of athletes at a major sporting event took place.^8^ This watershed study investigated the perceived impact of poor oral health on athletic performance and reported that elite athletes appeared to have more oral healthcare issues in terms of oral disease than an equivalent sample of the population.

Reports from previous Summer and Winter Olympic Games indicated that there was a substantial oral health treatment need for athletes and suggested that there may be high levels of oral health problems.^9^^,^^10^^,^^11^^,^^12^^,^^13^^,^^14^ The collection of oral health data of elite athletes is important:

- To understand the oral health of athletes and to determine how to implement relevant preventive care programmes^15^
- To inform the planning committees of dental services at future games including the scope of resources and skills needed by the dental workforce
- To collect relevant data as poor oral health negatively affects oral health-related quality of life.^16^^,^^17^

It has subsequently been shown that the link between athletic performance and oral health is compelling^18^ and that this could adversely affect athletic performance and training. Moreover, the link between poor oral health and systemic health has been well-documented.^19^

White, Giblin and Boyd (2017),^20^ reported that one in five patients exhibited anxiety about dental treatment and that if anxiety could be reduced there was a possibility of more regular attendance. However, there are no studies looking specifically at elite athletes and their levels of anxiety at visiting the dentist. This could be a contributing factor to poor attendance records from elite athletes.

## Planning facilities

Thorough planning of dental facilities at major sporting events is crucial^21^ from the perspectives of manpower, financial and risk management,^22^ ensuring the provision of appropriate care for athletes and associated members of the sports team. The local organising committee's planning decisions will determine the level of dental care incorporated within the medical set-up, ranging from immediate pain relief services to get any athletes out of pain, to a comprehensive dental care treatment option that is available to the athletes, their support teams and sometimes spectators. Additionally, a preventive programme may be included, where dental care professionals offer oral hygiene advice, dietary recommendations, and mouthguard production services, as athletes often forget or lack suitable mouthguards. If a mouthguard service is provided, the team should include a dental technician and the necessary equipment to quickly produce custom mouthguards and repair dentures and prostheses in cases of trauma.

**Fig. 1:**
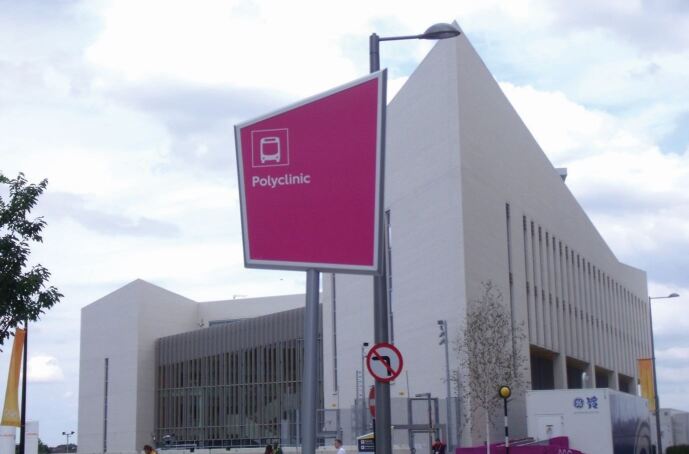
The Polyclinic at London 2012 (image courtesy of Professor I. Needleman)

**Fig. 2:**
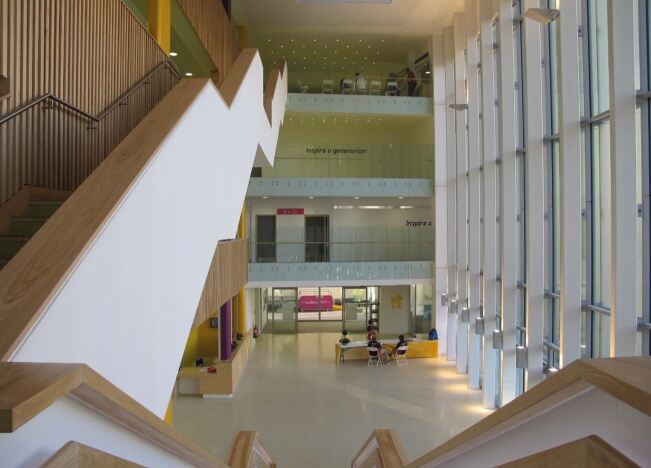
London 2012 Olympic medical facility with the dental facility on the top floor

The decision about the level of dental care provided is frequently made on financial grounds because the budget for dental facilities is often limited and needs to be part of the medical team's overall budget. Dental facilities would ideally be situated in a healthcare clinic alongside other clinical teams (Table 1), including physical therapies, sports medicine, imaging, podiatry, pharmacy, optometry, family practice, and emergency medical services (Fig. 1, Fig. 2). Dental facilities should be considered for paralympic athletes, who present with similar dental issues. These include accessible buildings, accessible surgeries, dentists who are familiar with treating patients with disabilities and an effective plan to return the injured athlete to the field of play (FoP). Medical services available at an Olympic Games polyclinic (with additional services at offsite location) can be seen in Table 1.

**Table 1:** Medical facilities typically found at major sporting events

Health Protection Agency Office
Sports medicine
Imaging (MRI, CT, xray, ultrasound)
**Dental services including field of play dentists**
Pharmacy
Primary care (family practice)
Laboratory services
Physiotherapy
Podiatry
Optometry
Emergency services
Overnight stay ward
Specialist clinics – e.g., dermatology
Hydrotherapy

A challenge for the dental team at major sporting events continues to be the logistics of equipping and servicing dental equipment at the dental facility. In addition to the arrangements to re-stock materials, any delivery of stock or attendance by engineers supporting the dental team need to have advance clearance to access the dental area, which is understandably protected by strict security measures.

The number of dental patients seen at the Olympic Games has risen since the Atlanta Games in 1996, where 906 dental patients were seen, peaking at London 2012, where 1,900 dental patients were seen; this represented 30 % of all cases seen in the medical centres. A further 1,000 patients were treated during the Paralympic Games at London 2012. To facilitate these numbers, the local organising committee needs to estimate the number of dental practitioners and dental care professionals (DCPs) who will be needed to support the athletes and team members likely to request dental care. In addition to dental treatment provided in the dental clinic, an estimate of how many specially trained ‘sports dentists' will be needed at various sporting venues, for example, boxing, hockey, rugby, and basketball, where orofacial trauma is relatively common.^23^ The need for these ‘sports dentists' to work in close proximity with the sport's medical teams at major sporting events, leads to a fully comprehensive service for elite athletes, while they concentrate on their performance. Estimating the number of dentists and specially trained ‘sports dentists' needed to cover an event is a function of the ‘medical and scientific commission', for example when it comes to an Olympic Games. They supervise the provision of healthcare and doping control at the games, deliver evidence-based education to athletes, develop and promote the adoption of ethical standards in sports science and medicine, explore the potential of new technologies in optimising athletes' health, and promote the health and physical activity of the entire population. The normal number of athletes attending a summer Olympic Games is in excess of 10,000, and it is estimated that approximately 70–75 dentists and DCPs are needed to volunteer, typically seeing over 100 patients per day during extended surgery hours so as to be as accessible as possible to athletes.

It is probably worth emphasising that the dental team attending Olympic Games are unpaid volunteers; although, some other major sporting events can involve contracting specialist sports dentists at a previously agreed rate. It is also important to recognise which days during the competition are going to be ‘peak days' and therefore more athletes are involved, so more medical/dental cover will be needed.

## Training the dental workforce

It is vital to actively encourage dentists to volunteer for these events, as their participation not only significantly enhances the support provided to athletes but also develops the presence of dental professionals at sporting activities. Providing comprehensive training for volunteers is very important, both before and during the event.^24^ The volunteer selection process must be thorough and equitable, ensuring that those chosen – whether medics or dentists – bring prior experience from similar events or showcase strong leadership abilities and an eagerness to learn how to effectively manage trauma in the FoP. In addition, new volunteers should be included, to gain valuable FoP experience. By fostering a dedicated and skilled volunteer base, we can truly transform the landscape of sports medicine and profoundly impact the lives of our athletes. Regular and frequent briefing sessions for the whole medical team are facilitated by senior medical staff.

The training needs to include familiarising:

1. The dentist with the environment they will be working in, which will undoubtedly be different to their normal dental practice and team they work with
2. Adapting to the limited resources they will have available to them
3. Discussing the level of care that will be delivered and to whom
4. Refresher courses delivered on common traumatic dental injuries seen on the sports field. ^21^

The training is analogous with training military dentists before deployment to the battlefield where they would be expected to resolve dental issues.^25^ Training should also include ‘risk assessment' and a knowledge of the laws of individual sports. For example, in rugby union, medical personnel can go onto the FoP to treat an injured athlete immediately, whereas in field hockey, they need to wait for the umpire to invite them on. To ensure the success of the event, it is crucial that the local organising committee takes charge of training volunteers for their specific dental roles. Dentists must be thoroughly familiar with their responsibilities, the facilities they will work in, their assigned teams, and their individual schedules. Additionally, dentists with specialist training are needed for some unique clinical situations faced by elite athletes, such as trauma and various oral health complications. Given the rarity of opportunities to conduct research with athletes, the chance to undertake meaningful research projects during this major sporting event should be considered very seriously.^26^^,^^27^

## The role of the dentist in the sports medical team

Dentists working at sporting events, whether the event is a regular professional club event or an international sports event, are part of the medical team, with special responsibilities for oral health issues, including dental trauma. If the organising committee include the provision of mouthguards within the role of the dentists, then this should be undertaken by the dental team. The dentists will be either based in the medical centre in the athletes' facility or at FoP. The FoP dentists should be encouraged to contact the lead medic and offer their services to the team, take part in any training opportunities, be willing to support medical colleagues during a match, be competent in dealing with orofacial trauma in a pressure environment and be supportive of any ongoing research. Those dentists working in the athletes' village (at an Olympic Games or world championship) should be competent at dealing with patients in pain, be able to quickly diagnose dental issues and provide support for the athletes, enabling them to continue with their event as soon as possible.^28^ As a volunteer, the dentists and DCPs should not exceed their level of competency and should not be afraid to refer an athlete for more specialist care if needed. To ensure support from their medical defence society, dentists and DCPs should avoid providing treatments they are not trained for or do not feel confident and competent in performing.

Dental clinics at sporting events can vary. Frequently, professional sports clubs do not have a specific dental surgery for treating athletes within their medical facility. So, the dentist finds themselves working in a physiotherapy room with minimal kit and treatment option. At major sporting events, very often an agreed number of fully equipped dental surgeries are planned as part of the medical facility. At London 2012, there were six dental surgeries available in the polyclinic, which became part of the legacy of the Games and are used to train dental students and provide dental care for the local community. At the Birmingham 2022 Commonwealth Games, three fully equipped mobile dental units situated throughout the city used by the Community Dental Service were available for athletes to visit for routine treatment, toothache and preventive advice. Table 2 illustrates some of the more common orofacial injuries seen by sports dentists at sporting events.

**Table 2:** Typical orofacial injuries seen at sporting events

**Type of injury**	**Details**	**Treatment**
Dental injuries	Fractured teeth Luxation injuries Avulsions	Dental repair Reposition tooth/teeth and consider splinting Replace tooth and splint
Injuries to the facial bones	Mandibular fractures Nasal fractures Zygomatic arch fractures Fractures of the orbit Temporomandibular Joint trauma	Attempt diagnosis and refer
Soft tissue injuries	Lacerations Ecchymosis	Clean wound and close Cold compress

With respect to FoP treatment, a decision is made by the local organisers on which sports require a sports dentist on-site in the event of an orofacial trauma. Clearly, sports like martial arts, boxing, rugby, hockey (field and ice) and basketball have a high potential for sports orofacial trauma and so are likely to require a dentist to be on-site. Other sports such as track and field, athletics, badminton and golf have a much lower incidence of orofacial trauma, so don't necessarily need a FoP dentist on site.^29^

## Examples of athletes treated for orofacial injuries

**Fig. 3:**
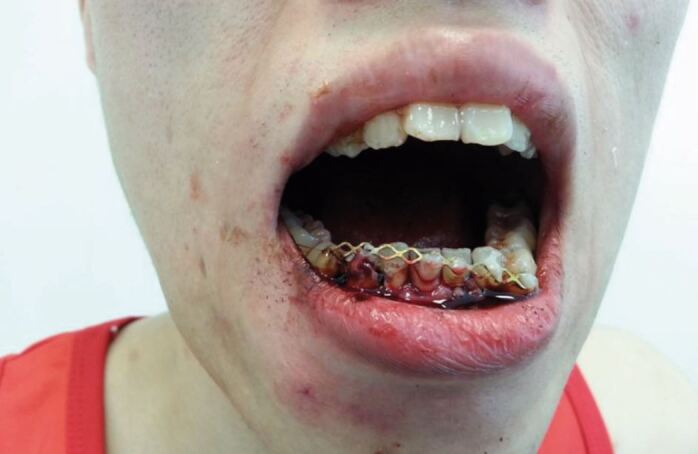
Splinted bi-lateral mandibular fracture of a kickboxer, using a titanium trauma splint

Figure 3 illustrates the use of a titanium trauma splint on a kickboxer following an illegal blow to his mandible, which resulted in him losing consciousness in the ring. A sports medic and sports dentist put him in the recovery position in the ring, checked his airway, breathing, circulation, disability and exposure before escorting him to the medical room. A bilateral fracture of the mandible, through the canine regions, was diagnosed by the sports dentist, necessitating immediate stabilisation of the anterior mandible before referral to a maxillofacial surgeon for a definitive repair. In this unusual case, the titanium trauma splint worked very well, considering the amount of bleeding and saliva in the field and the minimal equipment and facilities available to the sports dentist.

**Fig. 4:**
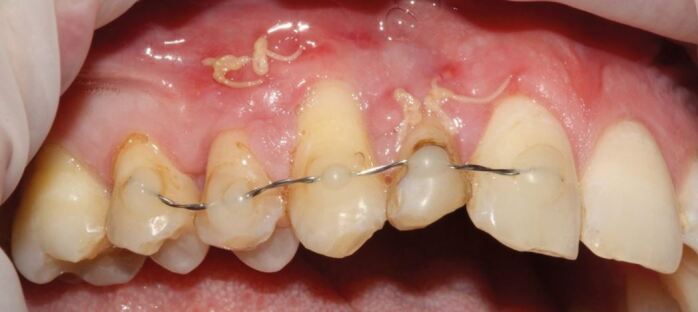
Avulsed upper right lateral incisor and canine, re-implanted and splinted on an international rugby player

Figure 4 illustrates the re-implanted upper right lateral incisor and canine tooth which were avulsed on this rugby player during an internation match. The player did not have a mouthguard in place, as he had previously reported that mouthguards felt uncomfortable. The splint illustrated in Figure 4 is a simple wire splint supported with composite, following the re-implantation of both fully formed teeth. Both teeth were subsequently root canal-treated, once the re-implanted teeth showed some degree of stabilisation following splint removal after two weeks. These teeth need to be monitored closely for five years to detect any consequential challenges, like root resorption and/or ankylosis. The initial splinting was carried out within an hour of the incident and soft tissue healing was promoted by some resorbable suture to approximate the edges of the wound.

**Fig. 5:**
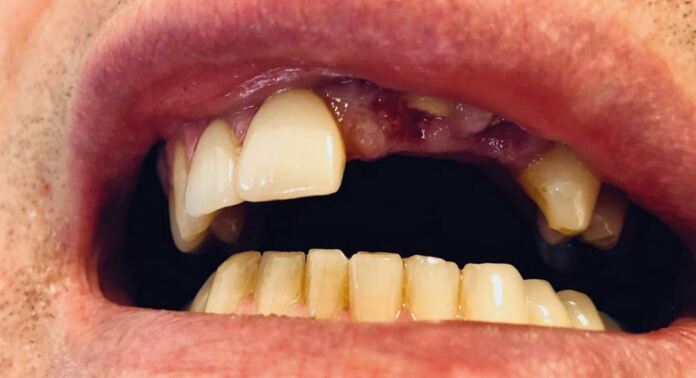
A 62-year-old recreational golfer who decoronated the upper left central and lateral incisors, while playing golf

Figure 5 illustrates the case of a 62-year-old recreational golfer after he was hit by a golf ball that re-bounded off a tree and de-coronated the upper left central and lateral incisors. Both teeth had previously been crowned with porcelain fused to metal crowns as had the upper right central and lateral incisors. Immediate care of this patient involved sealing the remaining roots with resin modified glass ionomer. It was impossible to use either of the previous crowns as there were completely shattered. A small removable partial denture was made, until a long-term solution had been agreed between the patient and their regular dental practitioner. The attending sports dentist was able to stabilise the situation, make the patient comfortable and looking respectable with the immediate spoon denture and offer advice about the sensible options going forward.

**Fig. 6:**
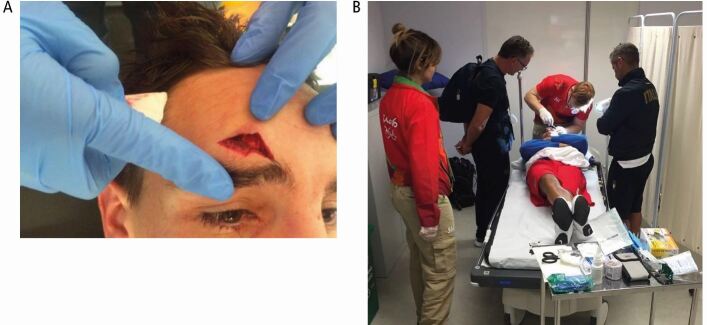
Example of a soft tissue injury in boxing and subsequent suturing

Figure 6 illustrates the kind of soft tissue injury seen during boxing, which was treated by a specialist sports dentist. This deep laceration needed full debridement of the wound and staged closure, using deep sutures to repair the muscle layer and then superficial sutures and surgical glue, to approximate the edges of the wound. Not only does this type of repair require great skill and practice but a real understanding of the anatomy of the skin and underlying musculature and the properties of the soft tissues. This requires lots of practice and a specialist knowledge, otherwise a very un-aesthetic outcome could result. It also requires knowledge of the individual sports regulations about allowing/not allowing the athletes to return to the FoP, once the wound is repaired. In boxing, a fighter cannot return to the ring with sutures visible or a bandage covering a recent trauma.

**Fig. 7:**
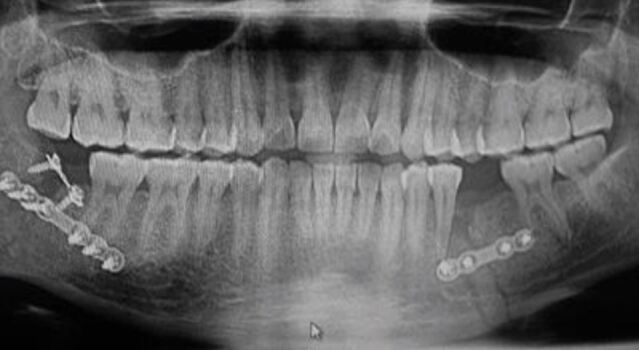
Radiograph of a fractured mandible following fixation

This boxer (Fig. 7) has been declared fit to box by his own medical team and presented to have a mouthguard made. After a clinical examination, the attending dentist was concerned about the level of recent injury previously disclosed and decided an orthopantomogram radiograph was needed. This special investigation revealed the extent of the injury and the necessary surgery to repair the mandible. The role of the dentist in this case was essential to prevent further trauma, which could have had serious implications if this patient had competed.

**Fig. 8:**
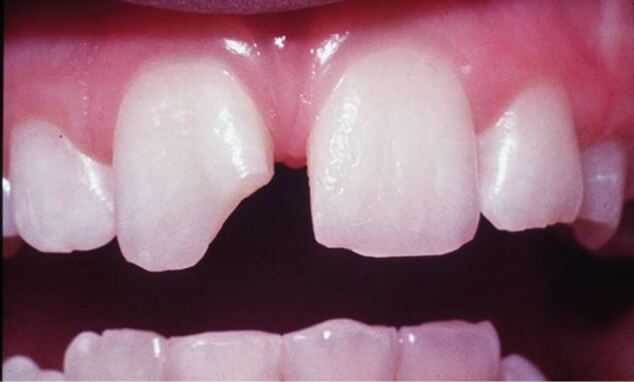
Uncomplicated tooth fracture in ten year-old soccer player

Figure 8 illustrates a typical uncomplicated crown fractured in a ten year-old soccer player. The sports dentist was able to cover the sensitive dentine with resin-modified glass ionomer cement before sending the patient to their regular dental practitioner. The fragment was found and stored in saline and was ultimately re-attached giving an aesthetic outcome, which was pleasing to the patient and parent.

## Supporting dental facilities at sporting events

**Fig. 9:**
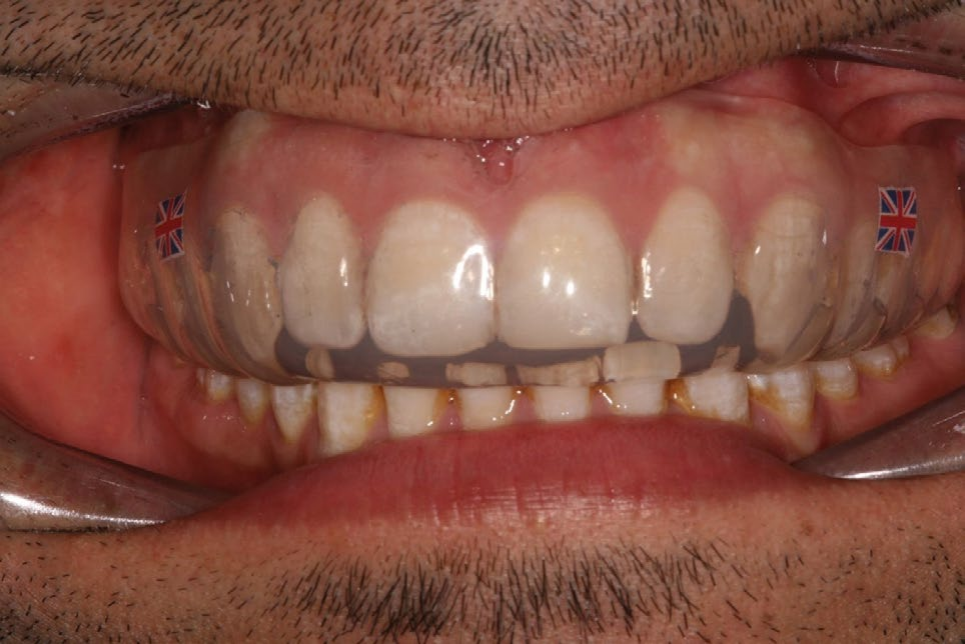
Example of a custom-made mouthguards made by on-site dental technicians during London 2012 Olympic Games

Apart from the obvious support from volunteer dental professionals at sporting events, other allied professionals also need to support the athletes. Dental technician facilities are needed if a mouthguard programme is going to be included as a preventive approach, for instance. Not all contact sports mandate the use of mouthguards, but boxing, for example, does require that all athletes wear a mouthguard. However, the legislation does not look at the quality of the mouthguard and so frequently these can be sub-standard and often cause soft tissue injuries^30^ (Fig. 9).

Support for medical and dental facilities at sports events is frequently seen in the shape of sponsorship from major global companies.^31^ Baim, Goukasian and Mosch (2021) reported a 0.44% impact on return, which amounted to a $61 million increase in sponsoring firms' market value. However, the event organisers need to be mindful of employing companies with positive health messages to be involved and not just those companies who offer the most money.

Support from other professions is also helpful to offer the visiting athletes the ‘complete package'. As well as dentistry we often see pharmacy, ophthalmology, sleep medicine, audiology and pathology services, many of whom donate materials and equipment in response for publicity.

## Summary

Providing medical care at sporting events can be both rewarding and challenging. Diagnosing and treating an athlete's toothache or managing orofacial trauma so they can continue competing is immensely satisfying. However, it can be frustrating when the clinicians rarely get to see the case through to its conclusion. Modern restorative materials allow dentists to create aesthetic restorations after dental injuries, but the expertise, knowledge, and confidence of sports dentists are crucial. The immediate initial restorative treatment provided at the polyclinic, or on the field, can have a major impact on the outcome for that athlete. Continuous training for sports dentists is essential to ensure they are prepared to handle any situation in the sporting arena, keeping both elite and recreational athletes safe and performing at their best capacity.
